# SGLT2 inhibitors and the underrecognized risk of euglycemic diabetic ketoacidosis: a clinical call for vigilance

**DOI:** 10.1097/MS9.0000000000003890

**Published:** 2025-09-15

**Authors:** Eisha Ali, Sheza Kamran, Asad Ali Ahmed Cheema

**Affiliations:** a FMH College of Medicine and Dentistry, Lahore, Pakistan; b International School of Medicine, International University of Kyrgyzstan, Bishkek, Kyrgyzstan

*To the Editor,*

This manuscript has been prepared in compliance with the TITAN Guidelines 2025 for transparency, integrity, and appropriate use of artificial intelligence in scholarly publishing^[1]^.

Sodium-glucose cotransporter-2 (SGLT2) inhibitors have gained momentum in clinical practice due to their striking cardiovascular and renal benefits in patients with type 2 diabetes, heart failure, and chronic kidney disease. Their widespread adoption is supported by consistent evidence demonstrating reductions in hospitalizations for heart failure and slowing of kidney disease progression, regardless of pre-existing atherosclerotic burden or renal function. However, amid this therapeutic enthusiasm, one critical safety concern remains underrecognized: euglycemic diabetic ketoacidosis (euDKA), a potentially life-threatening complication whose atypical presentation may escape detection in routine clinical care^[2]^. Real-world clinical experience indicates that euDKA frequently arises in the context of reduced insulin dosing, carbohydrate restriction, acute illness, or volume depletion particularly among patients receiving SGLT2 inhibitors^[3]^.

Unlike traditional diabetic ketoacidosis (DKA), euglycemic DKA (euDKA) is characterized by normal or only mildly elevated blood glucose levels, often resulting in delayed recognition and treatment. Multiple case series and pharmacovigilance reports have documented its occurrence particularly in patients undergoing perioperative fasting, adhering to low-carbohydrate diets, experiencing acute illness, or reducing insulin doses while receiving SGLT2 inhibitors. Because glucose levels may appear deceptively normal, frontline clinicians risk overlooking the diagnosis until severe metabolic decompensation has occurred. As emphasized in recent literature, the absence of marked hyperglycemia renders the clinical presentation of euDKA subtle, increasing the likelihood of delayed diagnosis and inappropriate initial management^[4]^.

The risk is especially pronounced in low- and middle-income countries (LMICs), where point-of-care ketone monitoring is often unavailable, and patients frequently present late with nonspecific symptoms such as malaise, nausea, or abdominal pain. In these settings, delayed recognition can result in significant morbidity, prolonged hospital stays, and avoidable healthcare expenditures. Limited access to ketone testing impedes early detection of DKA, exacerbates diagnostic delays, and places additional strain on already overburdened health systems^[5]^.

In summary, while SGLT2 inhibitors have revolutionized the treatment of diabetes and heart failure, awareness of euglycemic diabetic ketoacidosis (euDKA) is paramount to guide safe prescribing. Increased clinician awareness, anticipatory patient education, and guidelines tailored to context are needed urgently to prevent this underappreciated but dangerous complication.

## Data Availability

Not applicable – no new data were generated or analyzed in this letter to the editor.

## Research registration unique identifying number (UIN)

Not applicable – this letter to the editor does not involve human participants and is based on previously published literature.
